# Prediction of students’ awareness level towards ICT and mobile technology in Indian and Hungarian University for the real-time: preliminary results

**DOI:** 10.1016/j.heliyon.2019.e01806

**Published:** 2019-06-18

**Authors:** Chaman Verma, Veronika Stoffová, Zoltán Illés

**Affiliations:** a Department of Media and Educational Informatics, Eötvös Loránd University, Budapest, Hungary; b Department of Mathematics and Computer Science, Trnava University, Trnava, Slovakia

**Keywords:** Education, Computer science

## Abstract

An experimental study was conducted to predict the student's awareness of Information and Communication Technology (ICT) and Mobile Technology (MT) in Indian and Hungarian university's students. A primary dataset was gathered from two popular universities located in India and Hungary in the academic year 2017–2018. This paper focuses on the prediction of two major parameters from dataset such as usability and educational benefits using four machine learning classifiers multilayer perceptron (ANN), Support vector machine (SVM), K-nearest neighbor (KNN) and Discriminant (DISC). The multi-classification problem was solved with test, train and validated datasets using machine learning classifiers. One hand, feature aggregation with the train-test-validation technique improved the ANN's prediction accuracy of educational benefits for both countries. Another hand, ANN's accuracy decreases significantly in the prediction of usability. Further, SVM and ANN outperformed the KNN and the DISC in the prediction of awareness level towards ICT and MT in India and Hungary. Also, this paper reveals that the future awareness level for the educational benefits will be Very High or Moderate in both countries. Also, the awareness level is predicted as High and Moderate for usability parameter in both countries. Further, ANN and SVM accuracy and prediction time is compared with T-test at 0.05 significance level which distinguished CPU training time is taken by ANN and SVM using K-fold and Hold out method. Also, K-fold enhanced the significant prediction accuracy of SVM and ANN. the authors also used a STAC web platform to compare the accuracy datasets using T-test and ANOVA test at 0.05 significant level and we found ANN and SVM classifier has no significant difference in prediction accuracy in each dataset. Also, the authors recommend presented predictive models to be deployed as a real-time module of the institute's website for the real-time prediction of ICT & MT awareness level.

## 1 Introduction

Data mining often called knowledge discovery in database (KDD), is known for its powerful role in uncovering hidden information from large volumes of data [1]. Its advantages have landed its application in numerous fields including e-commerce, bio-informatics and lately, within the educational research which commonly known as Educational Data Mining (EDM) [2]. EDM is a budding discipline related with innovative methods for discovering the exclusive and increasingly big data that come from the educational background and using those methods to better understand to the stakeholders [3]. The fundamental principle of EDM is to analyses the educational data from different angles, categorize it and finally to summarize it [4]. The statistical analysis with F-test, T-test has been also used in the educational data mining field [3, 5, 6, 7]. But nowadays, EDM is also being a very popular area of research which uses machine learning and data mining techniques to explore more and more data from educational settings [8]. Machine learning is trending in the educational field for data mining purposes. In addition, machine learning is used to extract patterns and relationship between data elements in the large, noisy and messy datasets [9]. In supervised learning, we just train datasets with test and validate input with preconceived output, having the idea that there is a relationship between the input and the output. For this many machine learning classifiers are trending to classify the data patterns in various fields [10]. The Support Vector Machine (SVM) is a supervised learning model introduced for binary classification in both linear and nonlinear versions [11, 12]. SVM performs classification by constructing an N-dimensional hyperplane that optimally separates the data into the two categories [13, 14, 15]. With the use of boosting technique, ANN generates a sequence of models to obtain more accurate predictions which are also called the ensemble model [16]. The Binary logistic regression is confined to only 2 classes, but discriminant (DISC) analysis is best suited for the multi-classification problem. The linear discriminant analysis (LDA) makes predictions by estimating the probability that a new set of inputs belongs to each class. It is used for homogeneous variance-covariance matrices whereas Quadratic discriminant analysis is used for heterogeneous variance-covariance matrices. K-Nearest Neighbors (KNN) is a non-parametric, lazy learning algorithm which is most suitable for multiclassification problem as well. The objective is to learn a function (f):X ⇒ Y in which predictors f(x):X can confidently be predicting the corresponding target Y which is Awareness levels. The demographic features of teachers and students were predicted in Asian and European institutions using machine learning [15, 17, 18, 19, 20]. Also, many of researcher had worked on educational datasets using machine learning classifiers as well [21, 22, 23, 24, 25]. The supervised machine learning classifiers play a significant role in predicting the patterns for any real-time system. The presented predictive models may help in the development of the real-time ICT based prediction system to predict future awareness in stakeholders towards the use of the latest ICT and MT resources. It may also be beneficial in the prediction of the real-time benefits of ICT tools, techniques, and equipment to the students. The concept of using machine learning can be beneficial for real-time age [26] and real-time locality [18] prediction of University student and also a prediction of the nationality of European school's student in real time environment are also recommended by [27]. Further, automation of the real-time gender prediction of European school's principal with the help of Web-server was also suggested [28]. The presented awareness predictive models can be deployed online as the real-time module on the University websites to predict the attitude [29], behavior, willingness and ICT awareness in the university students with monitoring technological access the following: 1. Age wise monitoring of the student's attitude towards usability, availability, issues, and opportunities of the trending ICT and MT Resources in the universities. 2. The locality of students can also be monitored towards ICT and MT awareness in education at real time. 3. The faculty of study or department of study of students can also be monitored at real time as well. The responses of students may be recorded on real-time website of the university and the predictive models may be useful to predict the future attitude, awareness levels and demographic features of the students towards the technological access.

## 2 Materials & methods

### 2.1 Dataset preprocessing

A well-defined structured questionnaire is designed using Google Form to collect primary data samples with stratified random sampling. Therefore, the hybrid scaled prone questionnaire is developed with 46 attributes. A hybrid means 5 points Likert, Binary scale (yes or No), nominal, etc. A research instrument has five major sections. First section belongs to 9 demographic attributes, second section belongs to the Development-Availability (DA) with 16 attributes, third section relates to the Attitude (A) with 6 attributes, fourth section belongs to the Usability (U) parameter with 6 attributes and last section belongs to the Educational Benefits (Edu. Benf.) with 9 attributes. The participated students were studying either in bachelor, master and doctorate courses. Out of 331 students, 169 students belong to the Eötvös Loránd University of Hungary and 162 students belong to the Chandigarh University of India. Hence, initially, the primary dataset consists of 331 instances and 46 attributes which are related to the 4 major ICT parameters belong to the A, DA, Edu. Benf., and U. Out of 6, we have 4 subsets of the master dataset, 2 subsets belong to Indian University and 2 subsets belong to the Hungarian University. Later on, for the prediction for both countries, we aggregated subsets and framed 2 aggregate datasets, one for Indo-Hungarian usability and second for Indo-Hungarian educational benefits. In this paper, we focused on only two parameters such as Edu. Benf. and U. Hence, we divided the main dataset into the 6 subsets which are shown in Table 1.

**Table 1:** Datasets description.

Datasets	Indian Usability	Hungarian Usability	Overall Usability	Indian Edu. Benf.	Hungarian Edu. Benf.	Overall Edu. Benf.
Instances	162	169	331	162	169	331
Attributes	6	6	6	9	9	9
Missing values	3	0	3	3	0	3

In online mode, only 6 missing value are handled with Weka 3.9.1 tool ***ReplaceMissingValue*** filter [2]. Based on the self-reduction, we eliminated 9 features related to the demographic characteristics such as age, gender, locality, nationality, study level, faculty, university, affiliation status, and home country. Also, we removed 16 attributes belong to the DA parameter and 6 Attributes relate to attitude parameter. Hence, a total of 15 attributes are selected which belongs to the Edu.Benf. and Usability parameter only. Also, ***InfoGainAttributeEval*** filter is used with Ranker Search algorithm in Weka 3.9.1 tool to calculate the rank of the considered attribute. The ***InfoGainAttributeEval*** filter evaluates the worth of an attribute by measuring the information gain with respect to the class. InfoGain(Class, Attribute)=(H(Class) - H(Class/Attribute)) Where H represents the Entropy. The ranking of 9 attributes is considered by inputting full training set with a combination of ***InfoGainAttributeEval*** and Ranker Search Algorithms. The calculated ranks of the influential attributes are shown in Table 2.

**Table 2:** Influential attributes.

Usability	Rank	Educational Benefits	Rank
Software Use	0.523	Higher Quality Lesson	0.732
Prepare Exercises and Class Assignment	0.445	Sharing of Resources, Expertise, and Advice	0.719
online professional development	0.35	Learning Outside Campus	0.714
Online communication with teachers	0.318	Enriches Learning	0.711
Download/Browse Material	0.302	Up-to-date Learning Materials	0.709
Internet Use	0.261	Improve Analytical Skills	0.642
—		Learning by Doing Approach	0.633
—		Reliable and Un-interrupted Downloading	0.602
—		Online Tutorial	0.60

As our main focus on the prediction of awareness by making a new class named awareness level using the calculating mean of responses with respect to Edu. Benf. and U of ICT and MT in higher education of both countries. we framed five awareness level named Very High, High, Moderate, Low and Very Low for target datasets belongs to the U and Edu.Benf. The authors confirmed that all stakeholders have provided their consent to further performing experiments.

### 2.2 Dataset training, testing and validation

IBM partition node is very useful utility Which splits the data into training, testing, and validation sets of model building and testing its performance. The six datasets are trained with two techniques holdout and validation method separately and collectively as well. To predict the ICT awareness level towards ICT and Mobile technology, individual and aggregate datasets are trained with four supervised machine learning classifiers (DISC, ANN, KNN, and SVM) with splitting; the first one is the test-train method (Holdout) and the second one is the test-train-validate method (validation) separately. The test-train method is applied to three training ratio such as 50-50, 60–40, and 70–30. In test-train-validate method, four ratio of dataset testing such as 40-40-20, 50-30-20, 60-20-20, and 50- 20–30. The accuracy discard policy of auto classifier node for each classifier is set as less than 95%.

### 2.3 Classifiers used

The predicted models are trained and tested using 4 supervised machine learning algorithms with validation in IBM Modeler. The discard policy of auto classifier is set up as less than 95% accuracy and the out of total 08 machine learning classifiers, the auto classifier algorithm suggested 4 best models SVM, ANN, KNN and DISC for the individual and combined dataset. Therefore, to predict awareness level towards usability, we used multilayer perceptron (ANN) using the boosting technique. One hand, to predict awareness level towards educational benefits at individual datasets, we used ANN and SVM and other hand ANN is applied on the aggregation of datasets.

### 2.4 Performance measures

In a multi-classification problem, IBM analysis node provided vital performances metrics to evaluate the results of experiments. We applied the following measures: (a) Coincidence matrices: We used combined matrices reflects actual values by rows and predicted values are defined by columns. (b) Performance evaluation Index (PEI): It is a measure of the average information content of the model for predicting records belonging to that category. The accurate predictions for rare categories will earn a higher performance evaluation index than accurate predictions for common categories. (c) Accuracy: The percentage of accurately predicted awareness level counts of the student from overall prediction counts. (d) Error: The percentage of inaccurately predicted awareness level counts of the student from overall prediction counts. (e) Right: Counts the total no. of right predictions from overall values. (f) Wrong: Counts the total no. of wrong predictions (1-accuracy) from overall values.

## 3 Experimental

### 3.1 Experiments and results analysis for usability prediction

In this section we trained, tested and validate the usability datasets separately and jointly. We found only ANN classifier suitable for applying on datasets as compared to others. Further, to enhance the predictive models, boosting techniques is also applied with ANN which significantly improved the accuracy of each dataset. With the use of boosting the accuracy of models increased by 4%. Afterward, the results are analyzed using combined coincidence matrices.

**Fig. 1:**
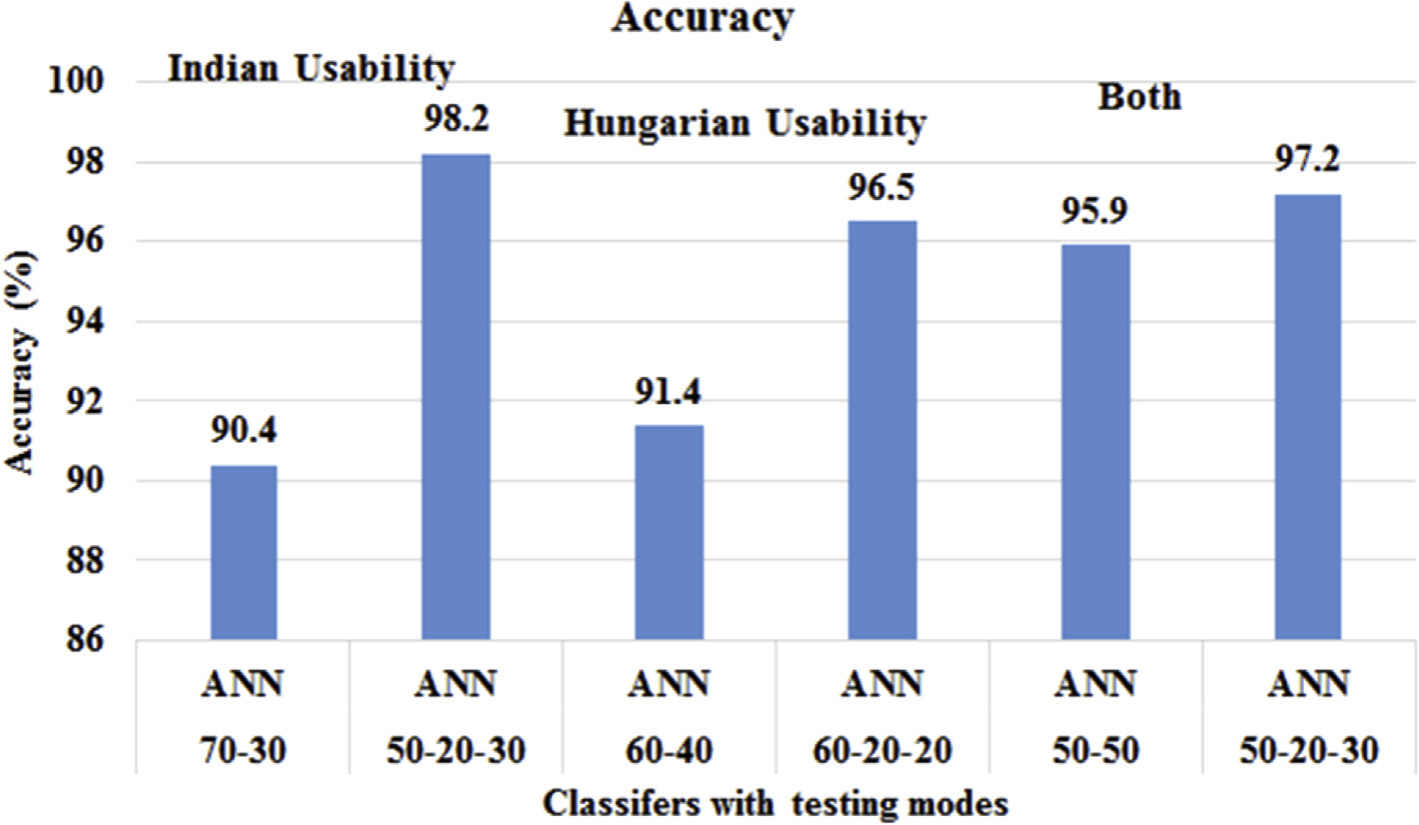
Usability Prediction Modeling using Test-Train and Test-Train and Validate method.

Fig. 1 displays the classifiers accuracy to predict the Usability of ICT and mobile technology individual and collectively in both countries. We found that out of 5 classifiers, only ANN fits for prediction task and on the training ratio 50-20-30, the highest accuracy is achieved as 98.2% in Indian usability. To predict the Usability in Hungarian universities towards ICT and mobile technology, the ANN classifier provided the highest accuracy of 96.5% at training ratio 60-20-20. The ANNs accuracy decreases down with training ratio 60–40. To predict overall Usability in Indian and Hungarian Universities towards ICT and mobile technology, ANN gained accuracy of 97.3% on training ratio 50-20-30. It is concluded that the accuracy increases with the validation approach of testing datasets with ANN.

**Fig. 2:**
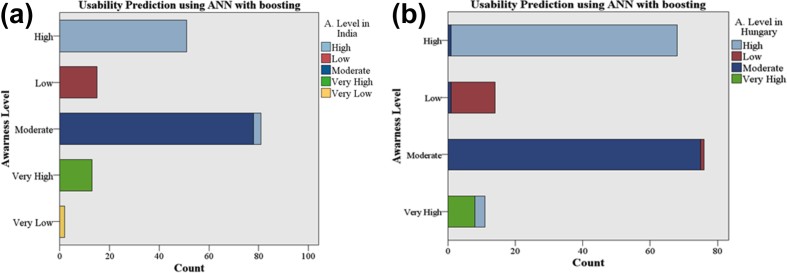
Usability prediction using ANN with boosting (a) of Indian students at 50-20-30 (b) of Hungarian students at 60-20-20.

Fig. 2 (a) shows the right prediction count of awareness level of High is 57, of moderate is 78, and of Very High is 13. The minor misclassification is found in the awareness level Moderate which is 3. Hence, it is concluded that we predicted the awareness level towards the usability of ICT and mobile technology is High, Moderate and Low in Indians students. Fig. 2(b) shows that at validation ration 60-20-20, the maximum awareness level is predicted as High (67) and Moderate (75). The prediction counts for Very High and Very low is calculated as 08 and 13 respectively. It is significantly found that the awareness level will be increasing as higher or moderate in Hungarian University's students. Therefore, ANN classifier significant evidenced that future awareness in the attitude of Hungarian students towards ICT and mobile technology in education will be higher or moderate likewise Indian students.

**Fig. 3:**
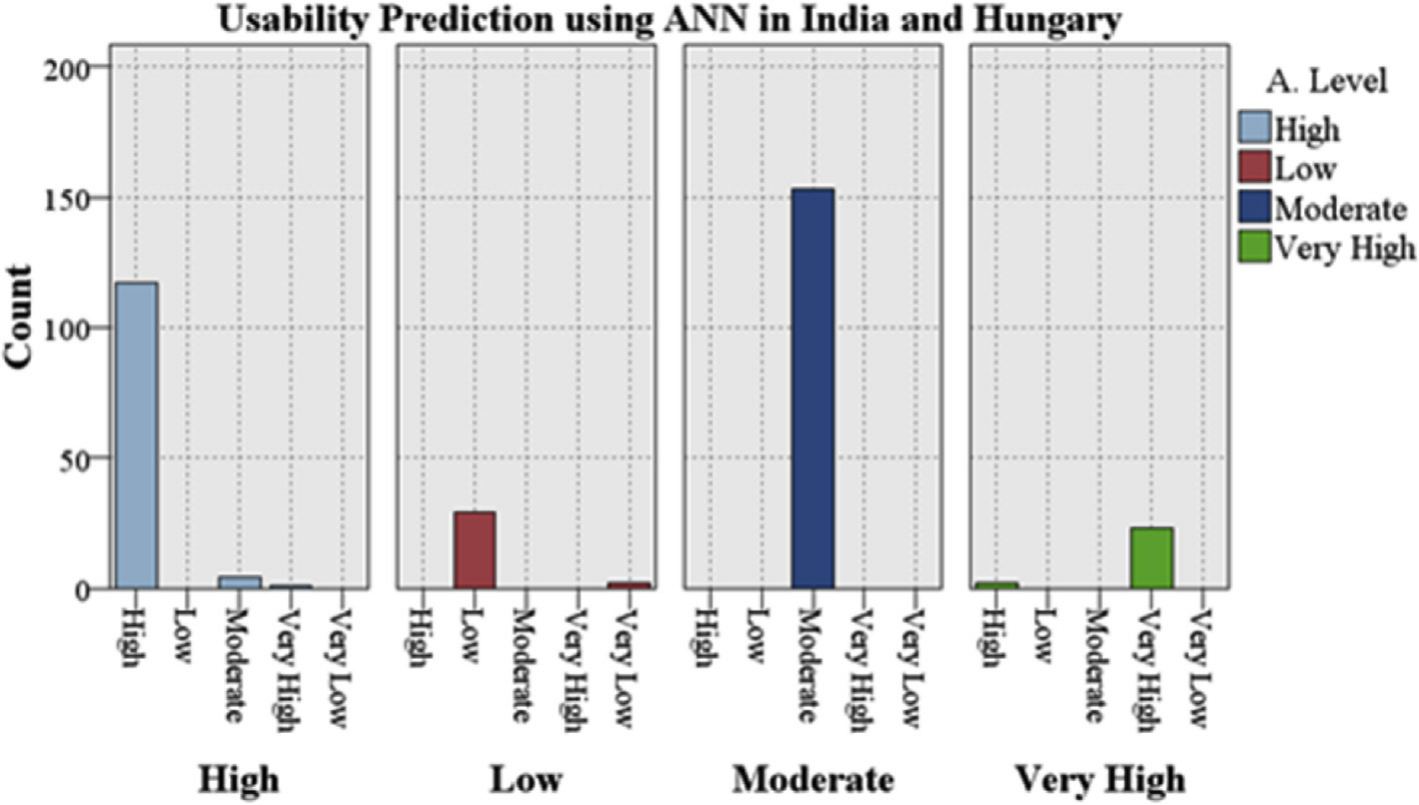
Overall Indo-Hungarian Usability Prediction using the ANN with a boosting at 50-20-30.

In Fig. 3 the combined testing approach stated that the Usability predictions for both of countries shall be High or Moderate as the ANN classifier gained the highest accuracy of 97.3% at training ratio 50-20-30 and the maximum count of awareness level for High is 117 and for the Moderate is 153. On combined datasets, the ANN also predicted an accurate count for the awareness level Very High (23). There is no significant misclassification is found in the prediction of Usability awareness level in both countries. Consequently, ANN proved that with the datasets aggregation increases the accuracy or prediction count with validation testing approach as compared to training ratio.

Table 3 shows the coincidence matrices belongs to the results provided by ANN with a boosting for the individual country's usability prediction. For Indian usability, the accurate count for the Very High, High, Moderate and Low is counted as 13, 51, 78 and 15 respectively at training ratio 50-20-30 with validation. Total no. of correct prediction is counted 159 out of 162. For Hungarian usability, correct prediction counts for the Very High, High, Moderate and Low is counted as 8, 67, 75 and 13 respectively at training ratio 60-20-20 with validation.

**Table 3:** Coincidence matrices for Individual usability prediction by ANN with a boosting.

Models		Indian Usability					Hungarian Usability			
Confusion Matrices		Prediction					Prediction			
A. Level		High	Low	Moderate	Very High	Very Low	High	Low	Moderate	Very High
Actual	High	51	0	0	1	0	67	0	1	0
	Low	0	15	0	0	0	0	13	1	0
	Moderate	3	0	78	0	0	0	1	75	0
	Very High	0	0	0	13	0	3	0	0	8
	Very Low	0	0	0	0	2	-	-	-	-

Table 4 shows the Overall predicted count for both of country's usability as 163 out of 169. Afterward, for both countries, the maximum predicted values counted for the Very High, High, Moderate and Low as 23, 117, 153 and 29 respectively at training ratio 50-20-30 with validation. It is evidenced that the incorporation of datasets with validation approach significantly raises the results of the awareness levels prediction for India, Hungary and both.

**Table 4:** Coincidence matrices for Joint usability prediction by ANN with a boosting.

Model		Indo-Hungarian Usability				
Confusion Matrices		Prediction				
A. Level		High	Low	Moderate	Very High	Very Low
Actual	High	117	0	0	2	0
	Low	0	29	0	0	0
	Moderate	4	0	153	0	0
	Very High	0	0	0	23	0
	Very Low	0	2	0	0	0

### 3.2 Experiments and results analysis for educational benefits

**Fig. 4:**
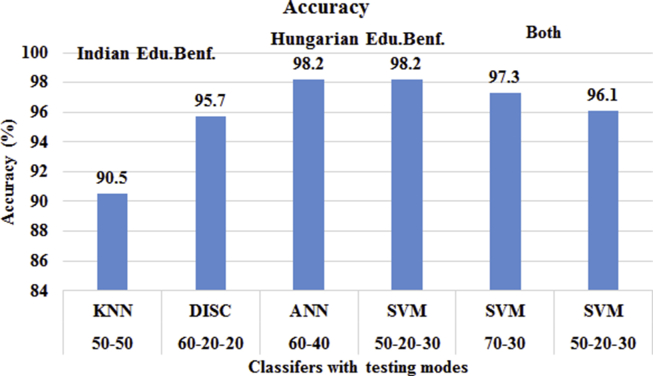
Educational benefits prediction modeling using test-train and test-train-validate method.

In this section, we trained, tested and validate the educational benefits datasets separately and jointly. We found KNN, ANN with a boosting and SVM classifiers are more suitable for these datasets. Further, the outcomes are analyzed using joint coincidence matrices. Data from Fig. 4 reflects no significant difference between ANN and SVM accuracy (98.2%) in the prediction of educational benefits for Hungarian students. We considered SVM with 50% training data, 20% test data and 30% validated data. In the case of Indian educational benefits prediction, DISC defeated KNN classifier in terms of accuracy. At training ratio 60-20-20, DISC gained 95.7% accuracy which is significant to the model. In the case of prediction for both of country's students, ANN with a boosting provided accuracy of 98.5% without validation sets and after validating datasets, the accuracy gets down by 0.9%. It is concluded that the accuracy decreases with the validation approach of testing joint datasets with ANN.

From Table 5 we can see results of SVM and DISC on three datasets of educational benefits. The DISC classifier predicted a maximum number of instances such as 67, 49,32 for High, Moderate and Very High respectively for Indian educational benefits. For Hungarian educational benefits, the accurate count for the Very High, High, and Moderate is counted as 42, 85 and 36 respectively.

**Table 5:** Coincidence matrices for Individual Educational benefits by DISC and SVM.

Models		Indian Educational benefits using the DISC				Hungarian Educational benefits using the SVM				
Confusion Matrices		Prediction				Prediction				
A. Level		High	Low	Moderate	Very High	High	Low	Moderate	Very High	Very Low
Actualaa	High	67	0	3	0	85	0	0	0	0
	Low	0	7	0	0	0	2	1	0	0
	Moderate	1	0	49	0	0	0	36	0	0
	Very High	3	0	0	3	2	0	0	42	0
	Very Low	-	-	-	-	0	0	0	0	1

Table 6 shows the Indo- Hungarian prediction, whereas overall accurate count is 325 out of 331 which proves the model is quite significant for deployment. For the Indo-Hungarian, the maximum predicted values are counted for the Very High, High and Moderate as 78, 155 and 86 respectively. Therefore, Multilayer perceptron (ANN) outperformed the SVM and DISC in the prediction of educational benefits to the students.

**Table 6:** Coincidence matrices for Joint Educational benefits by ANN.

Model		Indo-Hungarian Edu. benefits				
Confusion Matrices		Prediction				
A. Level		High	Low	Moderate	Very High	Very Low
Actual	High	155	0	0	2	0
	Low	0	6	3	0	0
	Moderate	0	0	86	0	0
	Very High	1	0	0	78	0
	Very Low	0	0	0	1	0

**Fig. 5:**
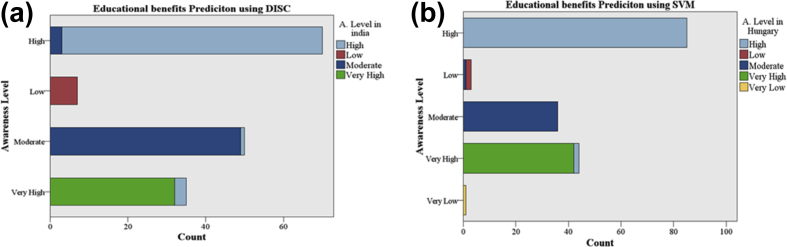
Educational Benefits prediction (a) using the DISC of Indian students at 60-20-20 (b) using the SVM of Hungarian students at 50-20-30.

Fig. 5 (a) shows the significant misclassification in level High and very High provided by DISC to predict educational benefits to the Indians students. The correct prediction count of awareness level of High is 67; of moderate is 49, and of Very High is 32 in Indians students. Fig. 5(b) shows that SVM achieved 100% classification for awareness level High and Moderate only. There is also minor misclassification (2) is also found in awareness level very High. Hence, it is concluded that future awareness level will be Very High, Moderate and High about consideration of educational benefits parameters. There is no possibility for awareness level will be of Low in Hungary. Also, DISC classifier proved the future awareness about educational benefits will be also High, Very High or Moderate in Indians students.

**Fig. 6:**
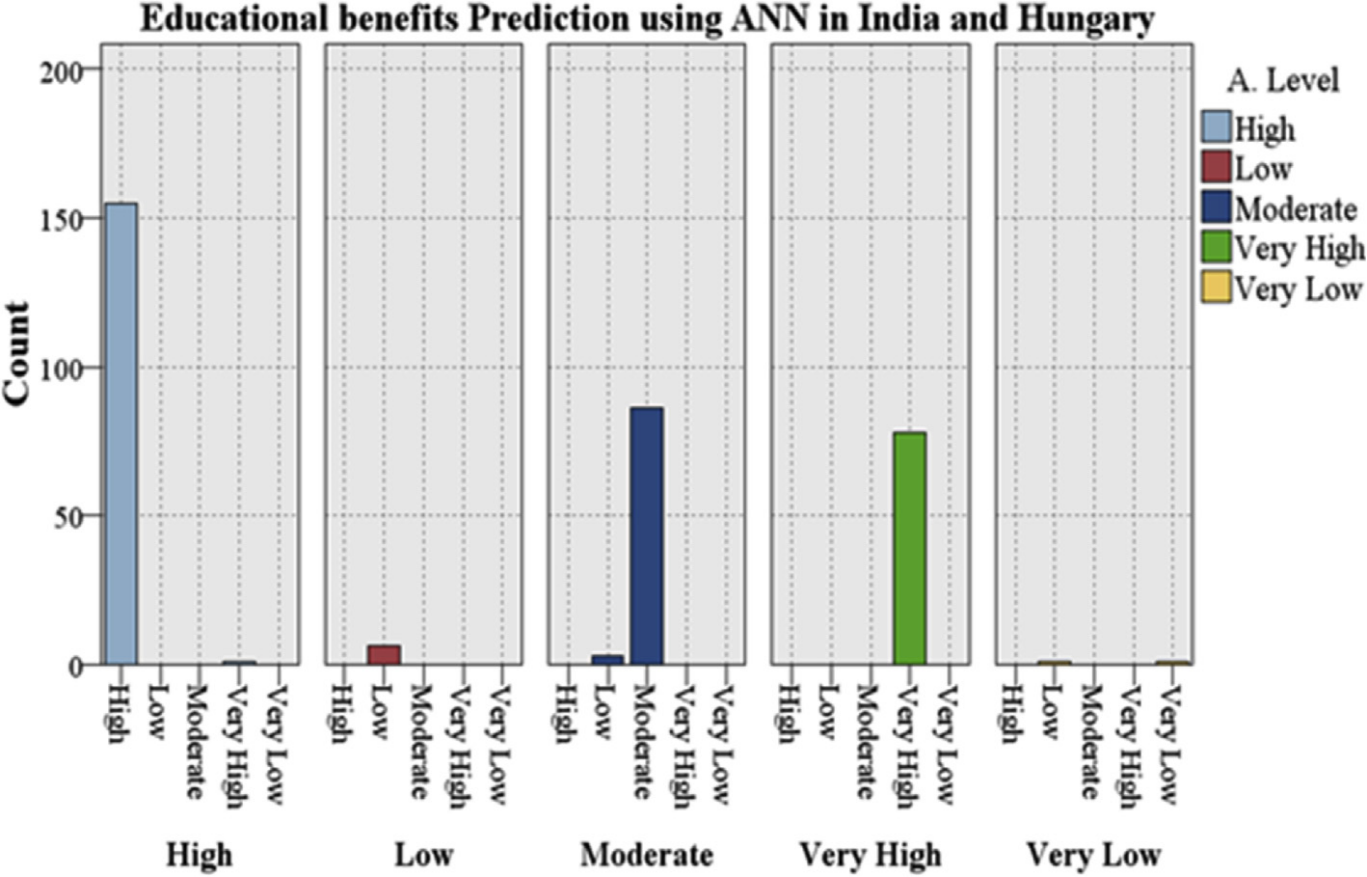
Overall Indo-Hungarian Educational Benefits Prediction using the ANN with a boosting at 70-30.

From Fig. 6 the awareness level towards Educational benefits of ICT and mobile technology in both countries will be High, Moderate, or Very High. In this combined testing approach, ANN with a boosting approach predicted the accurate count of awareness level for High is 155, for Moderate is 86 and for Very High is 78.

### 3.3 Experiments and results analysis for Prediction Accuracy and Time comparison

This section explores the results of experiments conducted using statistical T-test at 0.05 level of significance with Weka Experiment environment. To evaluate the performance of classification algorithms in terms of prediction accuracy versus CPU training time with the help of statistical analysis is significant and suggested [29, 30]. To present a real-time significant model, this experiment compared the induced User CPU time to predict the student's awareness level. For this, we have tested and validated 6 datasets separately using hold out method and K-Fold cross-validation with 10 iterations adoring with T-test at 0.5 significant level to keep in view two parameters named CPU Training Time (CTT) and Accuracy. The Hold out method used training ratio of 66:44 and K-fold cross-validation used 10-Fold cross-validation with k = 10 to enhance the prediction accuracy.

**Fig. 7:**
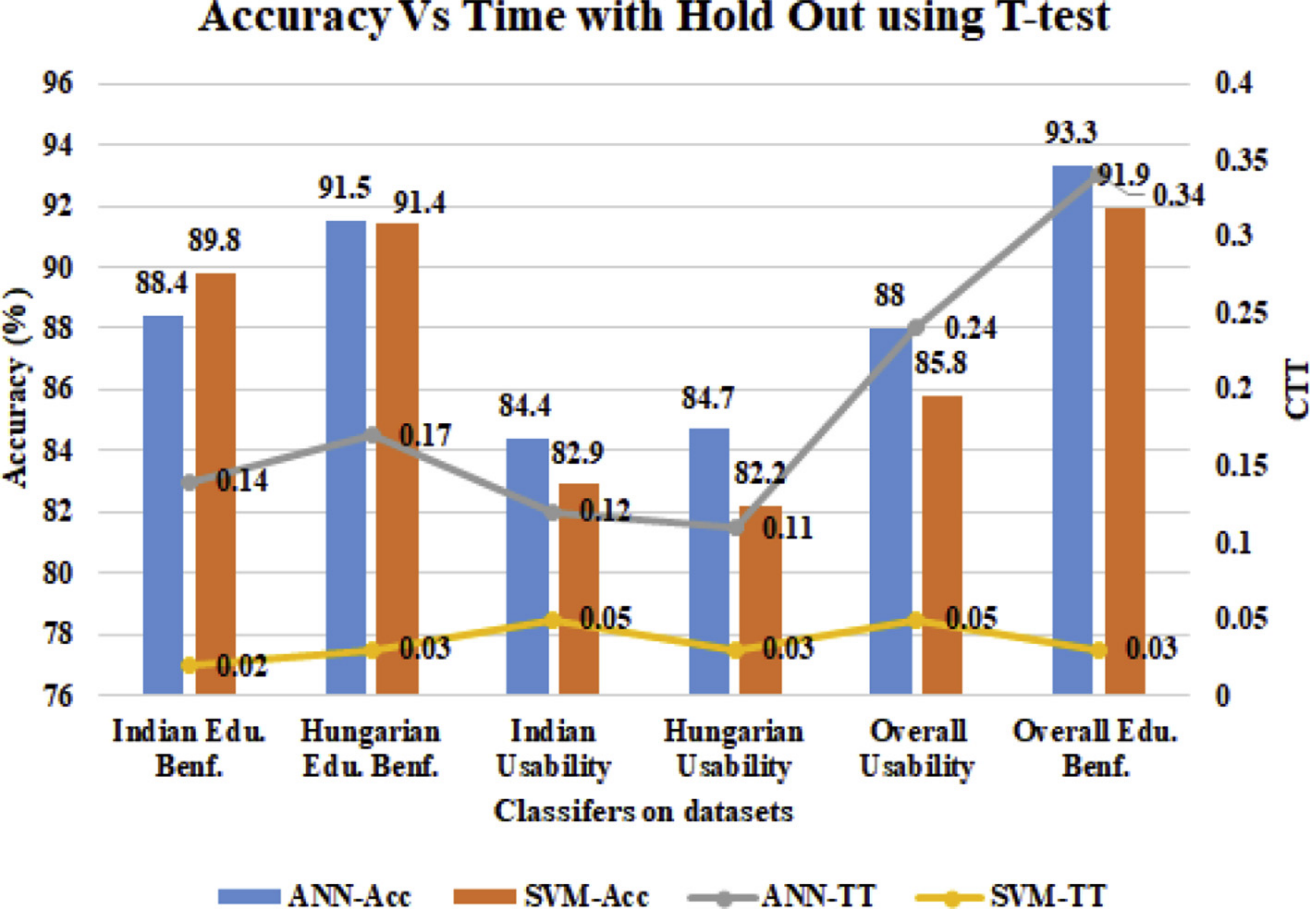
Real-Time Vs Prediction Accuracy using Hold out with T-test at 0.5 significant level.

In Fig. 7, the primary y-axis denotes accurate prediction accuracy of awareness level and the secondary y-axis shows CPU time in seconds. The x-axis shows the comparison of classifiers on 6 datasets. For Indian Edu. Benf. dataset, the SVM outperformed the ANN in prediction accuracy (89.8%) and in CTT (0.02 seconds). For Hungarian Edu. Benf. dataset, the ANN outperformed the SVM in prediction accuracy (91.5%). The ANN CTT is induced 0.14 seconds which is higher than SVM CTT. Also, ANN outperformed SVM in prediction accuracy on Hungarian U (84.7%) and Indian U (84.4%) dataset. In case of aggregate datasets (overall), ANN outperformed SVM in prediction accuracy with 88% in U dataset and 93.3% in Edu. Benf. datasets. It is also noted that ANN has induced higher CTT as compared to SVM in every case.

**Fig. 8:**
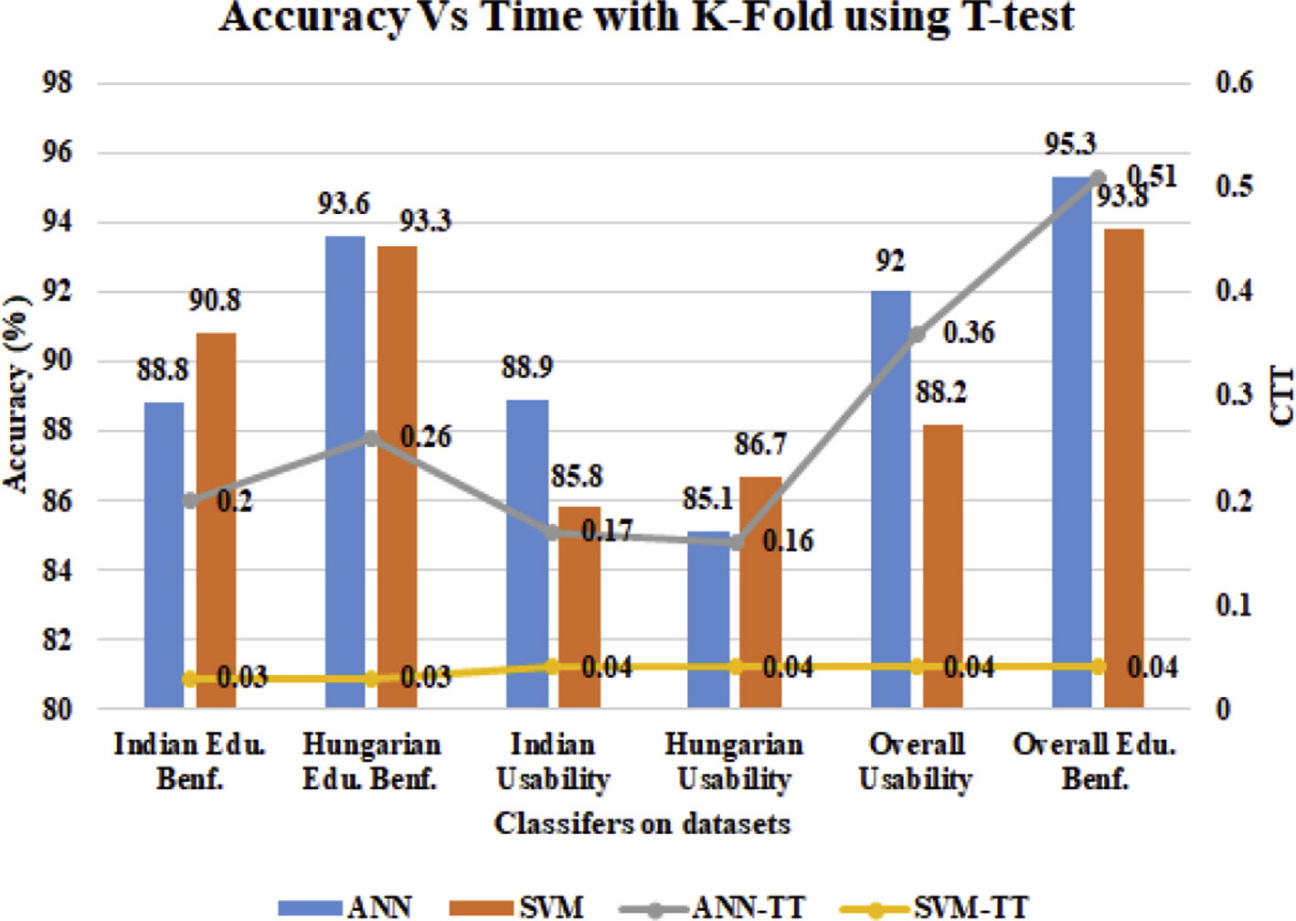
Real-Time Vs Prediction Accuracy using K-Fold with T-test at 0.5 significant level.

Fig. 8 shows the results produced using K-fold cross-validation testing methods with T-test at the 0.05 significance level. It is found that with k = 10, the prediction accuracy of SVM and ANN are enhanced as compared to the Hold out method previously shown in Fig. 7, One hand, SVM (90.8%) outperformed ANN (88.8%) on Indian Edu. Benf. dataset and another hand, ANN (93.6%) outperformed SVM (93.3%) in prediction accuracy. Also, it is found that for Hungarian U the SVM (86.7%) outperformed the ANN (85.1%) in prediction accuracy. In the case of Indian U dataset, ANN attained the highest accuracy (88.9%) as compare to SVM (85.8%). One hand, for the overall usability, ANN outperformed with 92% accuracy with SVM having accuracy with 88.2%. Another hand, the ANN has also outperformed the ANN in overall Edu.Benf. dataset with the increasing accuracy by 1.5%. In this experiment, it is also found that SVM's CTT is lowest as compared to ANN's CTT on each dataset.

Further, the STAC web platform [31] is also used to compare the performances of ANN and SVM classifiers on each accuracy datasets with holdout (60:40) and k-fold (k = 10) method. The normality of accuracy datasets is tested with the Shapiro-Wilk test [32] at a significance level of 0.05.

Table 9 shows the results of the Shapiro-Wilk test at the 0.05 significance level to find the normality of datasets. For this, the authors framed the first null hypothesis named “nH0: The samples follow a normal distribution". The authors did not found significant p-value using Shapiro-Wilk test at 0.05 level of significance at Hold Out (60:40) and K-fold method (k = 10). Therefore, the authors found that accuracy datasets are normally distributed.

**Table 9:** Shapiro-Wilk test at 0.05 significance level.

Classifier with Test Method	p-value	nH0
ANN (60:40)	0.960	accept
SVM (60:40)	0.257	accept
ANN (k = 10)	0.960	accept
SVM (k = 10)	0.433	accept

Subsequently, to test the homoscedasticity of accuracy datasets with the second hypothesis is framed as “hH0: All the input populations come from populations with equal variances”. Table 10 displays the results of the Levene test [33] at 0.05 significance level to find the homoscedasticity of the accuracy datasets. The authors found that all the input populations come from populations with equal variances. Hence, parametric t-test [31] is appropriate suitable to apply on accuracy datasets to compare the performances of machine learning algorithms. For this, the authors assumed the null hypothesis “aH0: No significant difference between the prediction accuracy of SVM and ANN”.

**Table 10:** Levene test at 0.05 significance level.

Test Method	Statistic	p-value	hH0
K-Fold (k = 10)	2.284	0.136	accept
Hold Out (60:40)	1.270	0.316	accept

In Table 11 we found the insignificant p-value for the null hypothesis aH0 at that at 0.05 significance level using the paired t-test. Hence, the null hypothesis aH0 is accepted which reveals that accuracy datasets of SVM and ANN have identical mean values. Hence. it is concluded that there is no meaningful difference is found between the prediction accuracy of ANN.

**Table 11:** T-test at 0.05 significance level.

Test Method	Statistic	p-value	aH0
K-Fold (k = 10)	0.870	0.424	accept
Hold Out (60:40)	1.763	0.138	accept

From Table 12, it is visible that we found the insignificant p-value for the null hypothesis gH0 at 0.05 significance level using paired ANOVA test. Therefore, the null hypothesis gH0 is accepted here. We found the means of the results of SVM and ANN prediction accuracy are the same. Hence, the ANOVA test also proved not any significant difference between the accuracy given by ANN and SVM classifiers.

**Table 12:** ANOVA test at 0.05 significance level.

Test Method	Statistic	p-value	gH0
Within Classifiers	3.413	0.028	accept
Between Classifiers	4.249	0.009	accept

## 4 Model

In this section, we evaluated the performances of the presented predictive models using various metrics shown in the combined Table 7 which displays the joint evaluation metrics of ICT awareness level predictive models with individual and aggregate features of the survey.

**Table 7:** Evaluation metrics.

Metric	Indian Usability ANN	Hungarian Usability ANN	Indo-Hungarian Usability ANN	Indian Edu. benefits DISC	Hungarian Edu. benefits SVM	Indo-Hungarian Edu. benefits ANN
Accuracy (%)	98.2	96.5	97.3	95.7	98.2	98.5
Error (%)	1.8	3.5	2.7	4.3	1.8	1.5
Correct	159	163	322	155	166	326
Wrong	3	6	9	7	3	5

The evaluation metrics showed the results in having more than 95% accuracy. To predict Indians usability, the ANN classifier with a boosting achieved the maximum accuracy of 98.2% with 1.8% error and the correct count of prediction is 159 and 3 are incorrectly predicted. The accurate count of the Hungarians usability and overalls usability prediction is counted as 96.5% and 97.3% respectively. Further, to predict educational benefits to Indian students, the DISC gained 95.7 accuracy and the correct count is found 155. Further, SVM obtained highest accuracy such as 98.2% for the prediction of Educational benefits to Hungarians and ANN scored 98.5% accuracy for the same prediction for overall respectively. It is concluded that SVM and ANN outperformed as compared to KNN and DISC in the prediction of awareness level towards ICT and mobile technology in India and Hungary.

Table 8 displays the PEI values for each class achieved by applied classifiers. One hand, for the rare category such as Very Low and Low, index values are found maximum by SVM and ANN in each dataset and other hands, for the common categories such as High, Very High and Moderate, the index values are lowest as compared to rare categories. But these values are found significant such as 1.5 and 1.3 for the class Moderate and Very High in Hungarian Educational benefits dataset. Although, for both countries, we also found 1.4 and 1.3 for Very High and Moderate receptively. Hence, it is proved that for both countries, future Educational benefits awareness levels shall be Very High or Moderate. Further, the Usability PEI values are also found significant such as 1.1 for High and 2.5 for Very High. For the Very Low class, we did not find any PEI values for Hungarian Usability, Indo-Hungarian Usability and Indian Educational benefits due to no values are found in their datasets.

**Table 8:** Performance evaluation index.

Metric	Indian Usability ANN	Hungarian Usability ANN	Indo-Hungarian Usability ANN	Indian Edu. benefits DISC	Hungarian Edu. benefits SVM	Indo-Hungarian Edu. benefits ANN
High	1.1	0.9	0.9	0.8	0.7	0.8
Low	2.4	2.4	2.4	3.1	4.1	3.5
Moderate	0.7	0.8	0.8	1.1	1.5	1.3
Very High	2.5	2.7	2.5	1.5	1.3	1.4
Very Low	4.4	-	-	-	5.1	5.1

## 5 Conclusion

The idea of testing various subsets and aggregate datasets with numerous type of classification algorithms at different training ratio provided better accuracy in the prediction of students ICT & MT awareness level in both countries. In the prediction of Indian U, boosting in ANN significantly improved the accuracy of each dataset. Hence, we presented three predictive models with maximum accuracy such as Indian U with 98.2%, Hungarian U with 96.5%, Overall U with 97.3%. Hence, it is also evidenced that the accuracy increases with the validation approach of testing usability datasets with ANN. One hand, in the prediction of Edu.Benf., we found no significant difference between ANN and SVM accuracy (98.2%) for Hungarian students and second hand, in the case of Edu.Benf. prediction, DISC beat KNN classifier in terms of accuracy. Further, it was also concluded that machine learning with validation and boosting technique improved prediction accuracy. It is also revealed that the educational benefits to both countries will be Very High or Moderate. Also, we did not find Very Low prediction for Indian usability and Hungarian educational benefits. Further, the awareness level is predicted as High and Moderate for the usability parameter in both countries. One hand, statistical T-test with Hold out and K-Fold method did not find a significant difference in between SVM's accuracy and ANN's accuracy in the prediction accuracy. Another hand, T-test found significant difference induced CTT in the prediction of each dataset. Also, K-fold method also enhanced significant accuracy of ANN and SVM as compared to Hold out method. Also, in the STAC web platform, the T-test and ANOVA tests also proved the insignificant difference between the accuracy of ANN and SVM classifiers on each dataset. Further, we recommend presented predictive models to be implemented as a real-time awareness level prediction of the university's student. Therefore, future work is also recommended for the creation of a real-time awareness prediction system using feature extraction with deep learning.

## Declarations

### Author contribution statement

Chaman Verma: Conceived and designed the experiments; Performed the experiments; Analyzed and interpreted the data; Contributed reagents, materials, analysis tools or data; Wrote the paper.

Veronika Stoffova: Analyzed and interpreted the data.

Zoltan Illes: Conceived and designed the experiments.

### Funding statement

This work was supported by European Social Fund under the project "Talent Management in Autonomous Vehicle Control Technologies (EFOP-3.6.3-VEKOP-16-2017-00001)".

### Competing interest statement

The authors declare no conflict of interest.

### Additional information

No additional information is available for this paper.
